# miRNA‐216 knockdown has effects to suppress osteosarcoma via stimulating PTEN

**DOI:** 10.1002/fsn3.1587

**Published:** 2020-08-09

**Authors:** Ping Jiang, Xin Yang, Yuanli Li, Juan Chen

**Affiliations:** ^1^ Department of Orthopaedics Affiliated Hospital of North Sichuan Medical College Nanchong China

**Keywords:** MG63, miRNA‐216, osteosarcoma, PTEN

## Abstract

The aim of this study is to explain the effects and mechanism of miRNA‐216 in osteosarcoma. We firstly evaluated the PTEN expression in 30 pairs of tumor and adjacent tissues which were from the 30 osteosarcoma patients. In the following cell experiments, we measured the cell proliferation, cell cycle, cell invasion, and migration abilities of NC (normal control) group, BL (blank) group, siRNA (miRNA‐216 inhibitor) group, and siRNA+PTEN inhibitor group. Furthermore, we measured the relative protein expression of difference groups by WB to explain the mechanism of miRNA‐216 in osteosarcoma. The PTEN was confirmed the target gene of miRNA‐216 by double luciferase target test. In conclusion, miRNA‐216 was an oncogene in osteosarcoma. miRNA‐216 knockdown had effects to suppress cancer cell proliferation, invasion and migration and improve cell apoptosis by keeping in G1 phase via PTEN.

## Graphical abstract

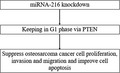

## 1 INTRODUCTION

Osteosarcoma is a highly aggressive bone tumor, which occurs in children and adolescents. It has high morbidity and mortality and seriously affects the quality of life of the patients (Lettieri et al., 2016; Rabinowicz et al., 2012). Conservative surgical resection is the main method for the treatment of primary bone tumors, although about 80% of conservative surgical resection can improve the survival rate of patients with osteosarcoma, but the patient's quality of life is greatly reduced, and the results of the study showed that surgical trauma could promote the dissemination and metastasis of malignant tumor tissues (Li, Liao, Xu, & Niu, 2016). Therefore, looking for a specific targeting gene for osteosarcoma can not only improve the quality of life of patients, but also inhibit the proliferation, invasion and metastasis of osteosarcoma.

miRNAs are a small, siRNA like molecule with a size of about 20–25 nucleotides (Jung & Suh, 2014; Tians, Wang, & Zhou, 2015; Wang, Zhu, Zhang, & Xiao, 2015). Encoded by the higher eukaryotes genome, miRNA is associated with target genes RNA‐induced silencing complex (RISC) degraded mRNA or hinder its translation. The mature miRNA is composed of longer primary transcripts by a series of nucleases cleaved, then assembled into the RNA‐induced RISC, by means of complementary base pairing of target mRNA, and according to the different guiding RISC degradation of target mRNA or inhibit translation of the target mRNA complementary degree (Onyido, Sweeney, & Nateri, 2016). mRNA, by binding to the ribonucleoprotein complex miRNP, recognizes the target mRNA and partially complements it, thereby thwarting the translation of target mRNA. In addition, miRNA can also cut completely complementary mRNA to degrade target mRNA (Jin & Lee, 2016). miRNA is involved in a variety of regulatory pathways, including development, viral defense, hematopoiesis, organ formation, cell proliferation, and apoptosis. Some previous studies found that miRNA‐216 was an important role in the cancer (Yang et al., 2013; Zhou et al., 2016).

Phosphatase and tensin homology deleted on chromosome ten (PTEN) is a tumor‐suppressor gene with phosphatase activity. The abnormal expression of PTEN protein has been confirmed in malignant gliomas, cervical cancer, and liver cancer tissues (Li & Sun, 1997; Li et al., 1997; Steck et al., 1997). We determined that PTEN was the target gene of miRNA‐216 by TargetScan software (http://www.targetscan.org/mamm_31/). In our present study, we firstly evaluated PTEN protein expression and HE staining of cancer and adjacent tissues; furthermore, we investigated the miRNA‐216 knockdown had effects and mechanism to osteosarcoma cell line MG63 in vitro study.

## 2 MATERIAL AND METHODS

### 2.1 Clinical data

Thirty pairs of tumor and adjacent normal tissues were collected from osteosarcoma patients who were treated in our hospital. The specimens were fixed with 10% formaldehyde solution and embedded in paraffin and made into thick 4‐µm slices. Immunohistochemical staining of PTEN was performed with biotin avidin biotin staining (S‐P). The cancer and tumor tissues were evaluated by HE staining. The relative kits were purchased from Beijing Zhongshan Biotechnology Co., Ltd.

### 2.2 Cell culture, transfection and grouping

The human osteosarcoma cell line MG63 was purchased from ATCC. The MG63 were cultured in DMEM (Sigma) contained with 10% fetal bovine serum (FBS) (Sigma), and the cells were cultured in the constant temperature cell incubator (37°C, 5% CO_2_). The MG63 changed the culture medium every days, Digestive passage by pancreatin at 3–5 days. At the time of transfection, the logarithmic growth phase cells were inoculated at six holes and the cells were grown to 50%–60%. The fusion 12 hr was synchronized with serum‐free medium and then transfected. The cells were divided into four groups: normal control (NC) group; blank (BL) group; simRNA transfection (simRNA) group; and simRNA and PTEN inhibitor transfection (simRNA+PTEN inhibitor) group. The simRNA and PTEN inhibitor were purchased from Guangzhou Ryde Biotechnology Co., Ltd. 20 µmol/L BL vector and 20 µmol/L simRNA‐216 or PTEN inhibitor were dissolved in MEM culture medium and mixed for 5 min at room temperature, other liposomes were added to Opti‐MEM medium temperature and mixed 5 min and then gently mixed to room temperature for 20 min. The mixture was added to the cells of the corresponding group, cultured in the incubator, replaced with 6 hr, replaced with normal cells, and the medium continued to culture 48 hr. The cell protein was extracted, and the transfection efficiency was determined and analyzed by subsequent experiments.

### 2.3 The cell proliferation by CCK‐8

Logarithmic growth phase cells were seeded in 96‐hole plates (1 × 10^4^ cell/ hole), according to the requirements of each treatment after cultured for 24 hr, per hole adding 10 μl CCK 8 reagent (Abcam), then continued to incubate for 30 min at 37°C. The absorbance (A) values of each hole at 450 nm wavelength were measured by microplate reader. Each group was divided into five double holes to take the average value, and only the medium hole was added as blank control. The experiment was repeated six times.

### 2.4 The cell apoptosis by flow cytometry

The MG63 cells in each logarithmic phase of growth were inoculated in the 6‐hole plate as 5 × 10^4^ cells/ml, adding 2 ml into each pore. The cells were cultured at 37, 5% CO_2_ and saturated humidity incubator for 24 hr. Each group has three holes. The cells digested with 0.25% trypsin, centrifuged at 13.5 cm, 800 r/min, centrifuged 5 min, and washed with PBS two times. In accordance with the instructions of Annexin V‐FITC/PI apoptosis detection kit (Sigma), the apoptosis was observed by flow cytometry, and the apoptosis rate was calculated automatically by cell quest software. The experiment was repeated three times and the mean value was taken.

### 2.5 The cell cycle by flow cytometry

The MG63 cell of difference groups which were treated by difference methods were cultured for 48 hr. Floating and adherent cells were collected, fixed with 70% ethanol, and subjected to PBS washing and centrifugation, adding 1 mg/ml RNase A and 50 g/ml PI (Sigma) and 30 min, reaction staining, and flow cytometry were collected and analyzed by the software Modfit cell cycle distribution.

### 2.6 The cell invasion by transwell assay

The MG63 cells of four groups which were in logarithmic growth phase were taken, and 2.5 × 10^5^ cell/ml single cell suspensions were prepared by serum‐free DMEM medium. Two hundred microliter was added to the upper chamber of Transwell, and DMEM containing 10% FBS was added to the lower chamber. Each group cells were cultured in the incubator for 24 hr, 4% paraformaldehyde fixed 20 min, with a cotton swab gently wipe the upper compartment of the cell, 0.1% crystal violet staining, inverted microscope magnified 100 times under five fields were counted and photographed. The experiment was repeated three times. The transwell bedroom was purchased from GREINER Company.

### 2.7 The cell migration by wound healing

Taking MG63 which were logarithmic were inoculated in 6‐well plates as 2 × 10^4^ cell/hole. When the confluence rate of each cell reached 70%, use a 10‐μl pipette head to make a “‐” on the six hole plate culture dish. PBS (0.01 mol/L) washed three times to remove floating cells, four groups of cell culture dishes, each group added 2 ml serum‐free medium, photographed under the inverted microscope, recording the initial scratch width value. The MG63 of four groups were cultured for 48 hr, cell migration was observed under an inverted microscope, and the scratch width of the corresponding time points was measured.

### 2.8 The relative proteins expression by WB assay

The MG63 cells of four groups were collected. The total protein was extracted by routine method, and the protein concentration was determined by BCA methods. After boiling denaturation, take the same amount of protein like behavior, SDS‐PAGE, pass through the membrane, room temperature 5%, milk closed, after adding antibody PTEN, PI3K, AKT, P53, MMP‐2, and MMP‐9 (Abcam) to incubate overnight. Rewarming after 30 min TBST rinsed four times (10 min/times), and horseradish peroxidase labeled two anti‐incubated for 1.5 hr, TBST after washing the membrane after using ECL chemiluminescence method using Quantity One 4.6.2 software developing image analysis. GAPDH was a reference in this experiment.

### 2.9 Dual luciferase assay

The use of Lipofectamine 2000^®^PTEN 3′‐UTRmiRNA reports plasmid and TK plasmid and miR‐216 pCDH expression vector or empty vector were co‐transfected into MG63 cells, 24 hr, determination of firefly luciferase and Renilla luciferase activity using FLUOstar Omega, and Renilla luciferase activity was used as a reference. Dual‐Lucy Assay Kit was purchased from Beijing Solarbio Science & Technology Co., Ltd.

### 2.10 Statistical analysis and methods

The relative data were analyzed by SPSS 19.0 software, and the results were expressed by Mean ± Standard deviation (mean ± *SD*), comparison of the data between groups was used by *t* test; *p* < .05, the difference was statistically significant.

## 3 RESULTS

### 3.1 The clinical data in the osteosarcoma patients

**FIGURE 1:**
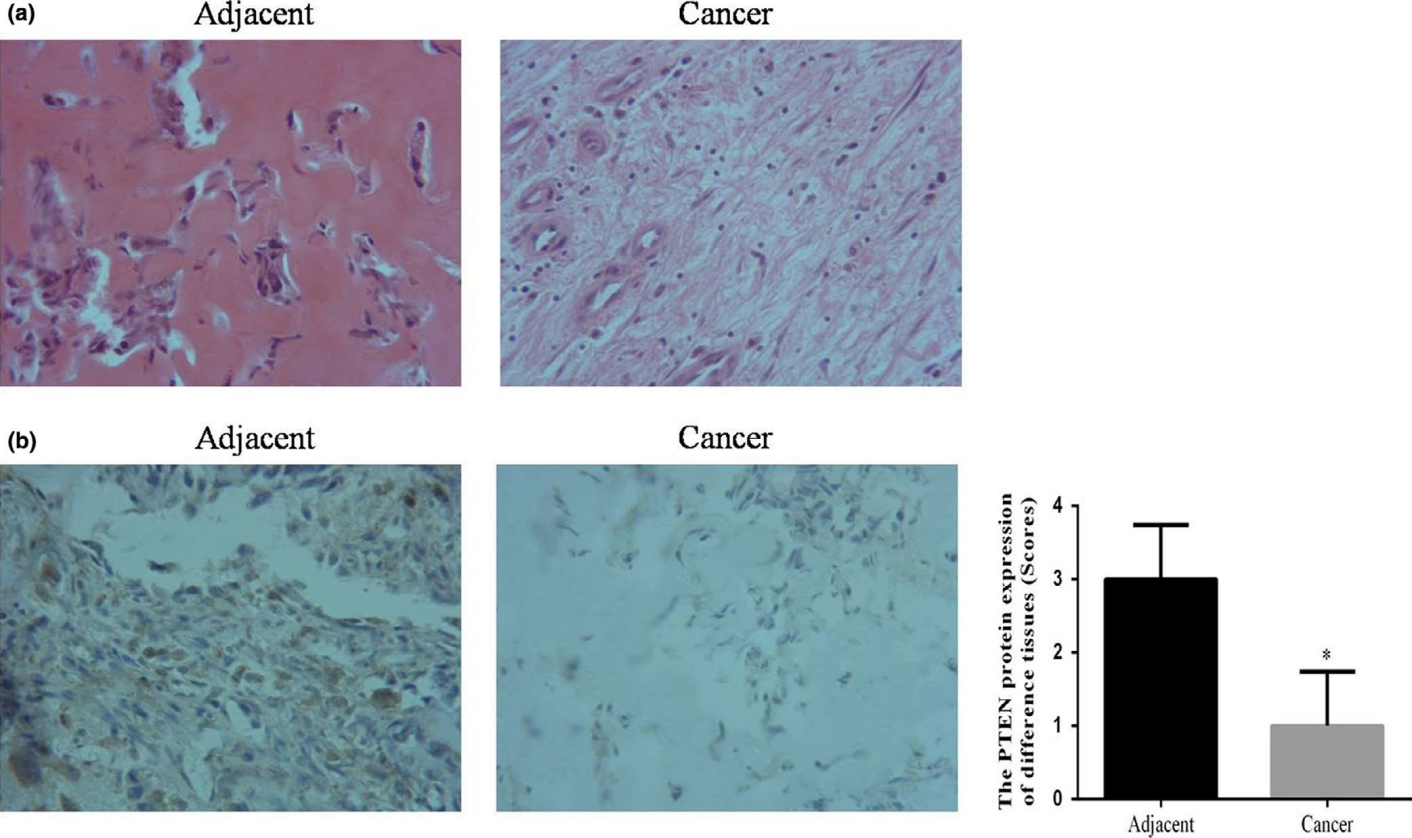
The clinical data. (a) The pathology of adjacent and cancer tissues by HE staining (×200). (b) The PTEN protein expression of adjacent and cancer tissues by IHC assay (×200). *p < .05; compared with adjacent normal tissues

The cell invasion ability of osteosarcoma tissues was enhanced compared with that of adjacent normal tissue by HE staining; the relative data are shown in Figure 1a. The PTEN expression of adjacent normal tissues were significantly up‐regulated compared with that of osteosarcoma cancer tissues by IHC assay (*p* < .05). The data are shown in Figure 1b.

### 3.2 The cell proliferation of difference groups by CCK‐8

**FIGURE 2:**
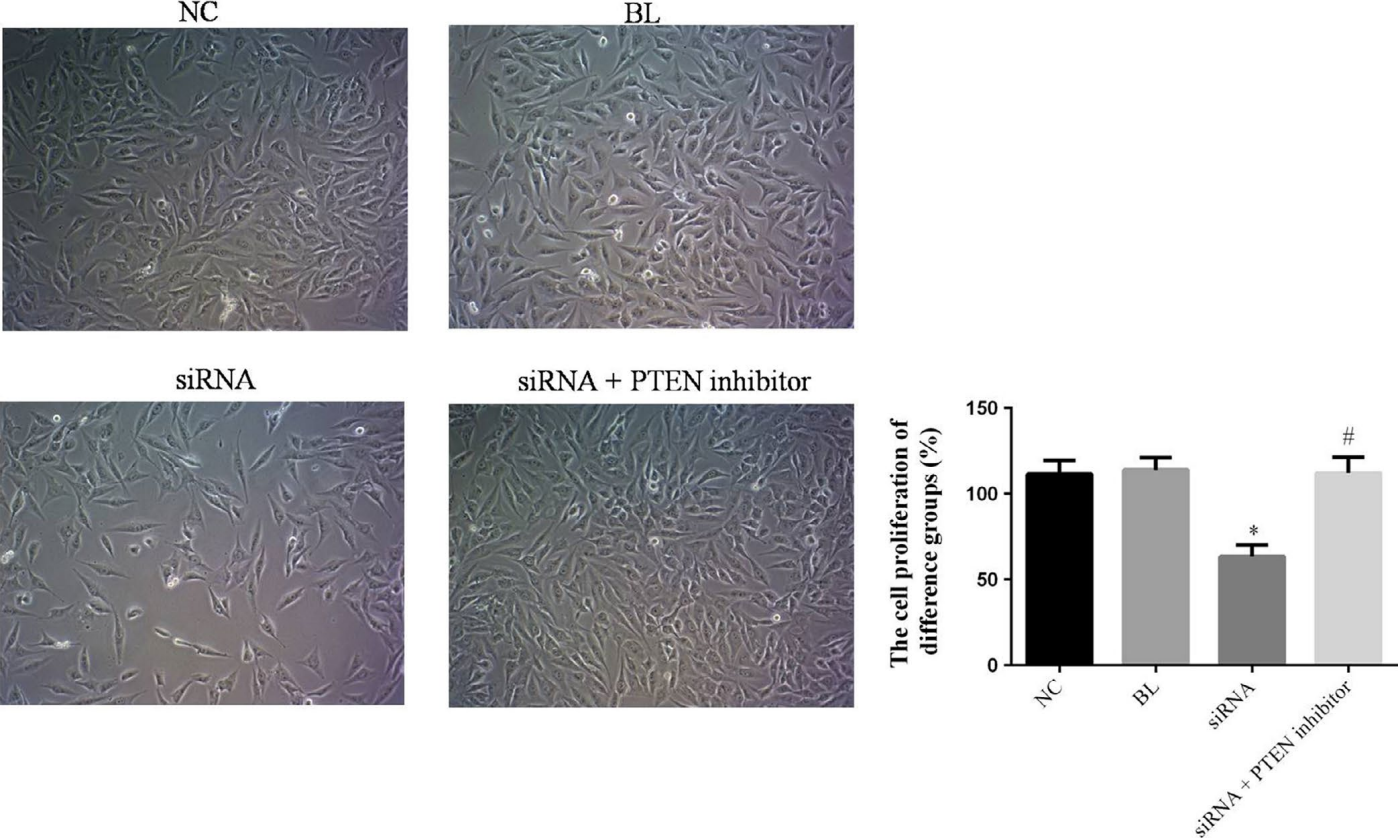
The cell proliferation rate of difference groups by CCK‐8. *p < .05; Compared with NC group. ^#^ < .05; compared with simRNA group

To investigate simRNA‐216 effects to cell proliferation in MG63, we measured the cell proliferation of different groups by CCK‐8. The results were shown that the cell proliferation of simRNA group was significantly suppressed compared with NC group (*p* < .05); however, the cell proliferation of simRNA+PTEN inhibitor group was significantly enhanced compared with simRNA group (*p* < .05) and no significant differences compared with NC group (*p* > .05). The relative data are shown in Figure 2.

### 3.3 The cell apoptosis of difference groups by flow cytometry

**FIGURE 3:**
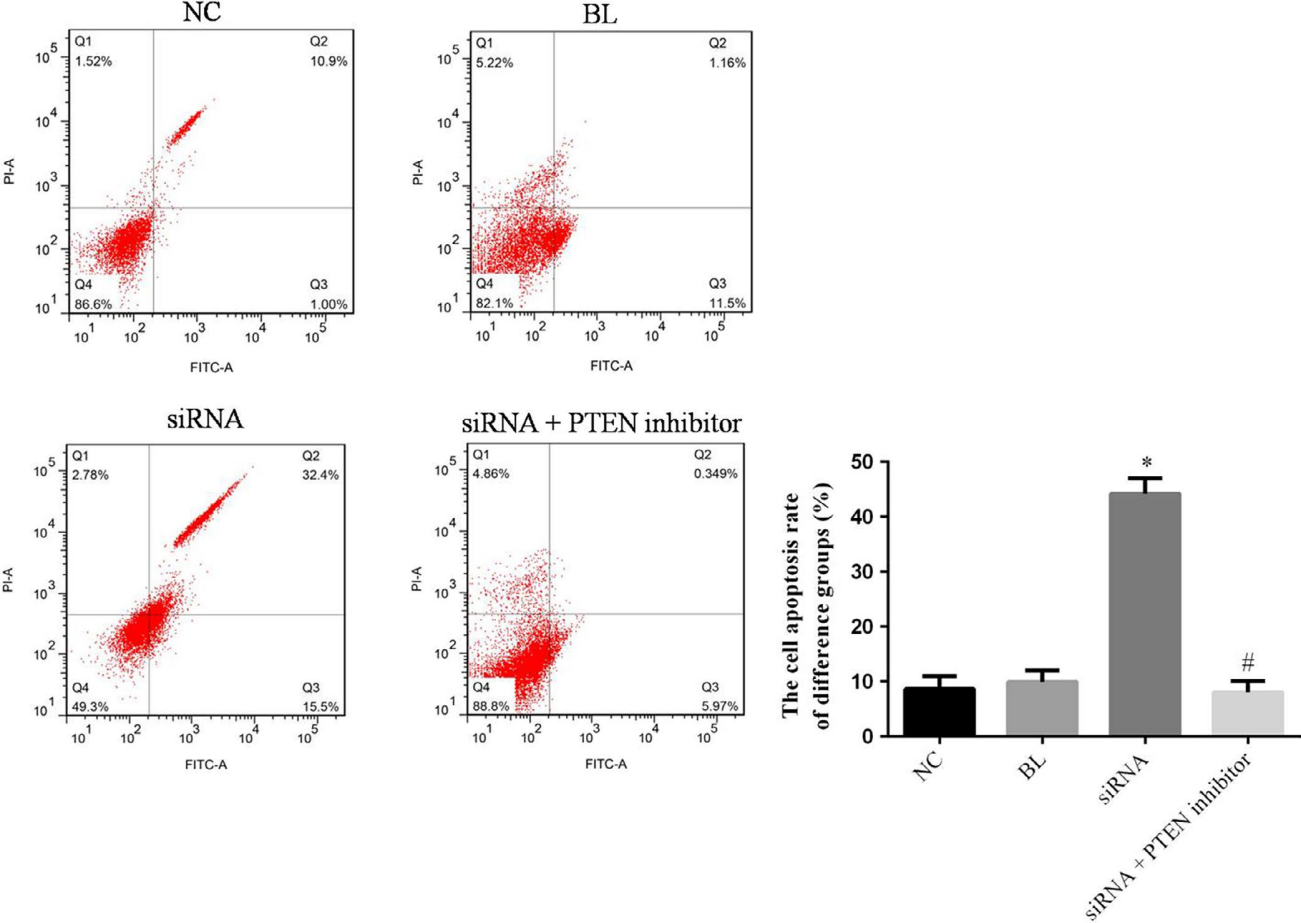
The cell apoptosis rate of difference groups. *p < .05; compared with NC group. ^#^ *p* < .05; compared with simRNA group

Compared with NC group, the cell apoptosis rate of simRNA group was significantly up‐regulated (*p* < .05), and the simRNA+PTEN inhibitor was no significantly differences (*p* > .05). The cell apoptosis rate of simRNA+PTEN group was significantly down‐regulated compared with siRNA group (*p* < .05). The relative data are shown in Figure 3.

### 3.4 The simRNA has effects to cell cycle

**FIGURE 4:**
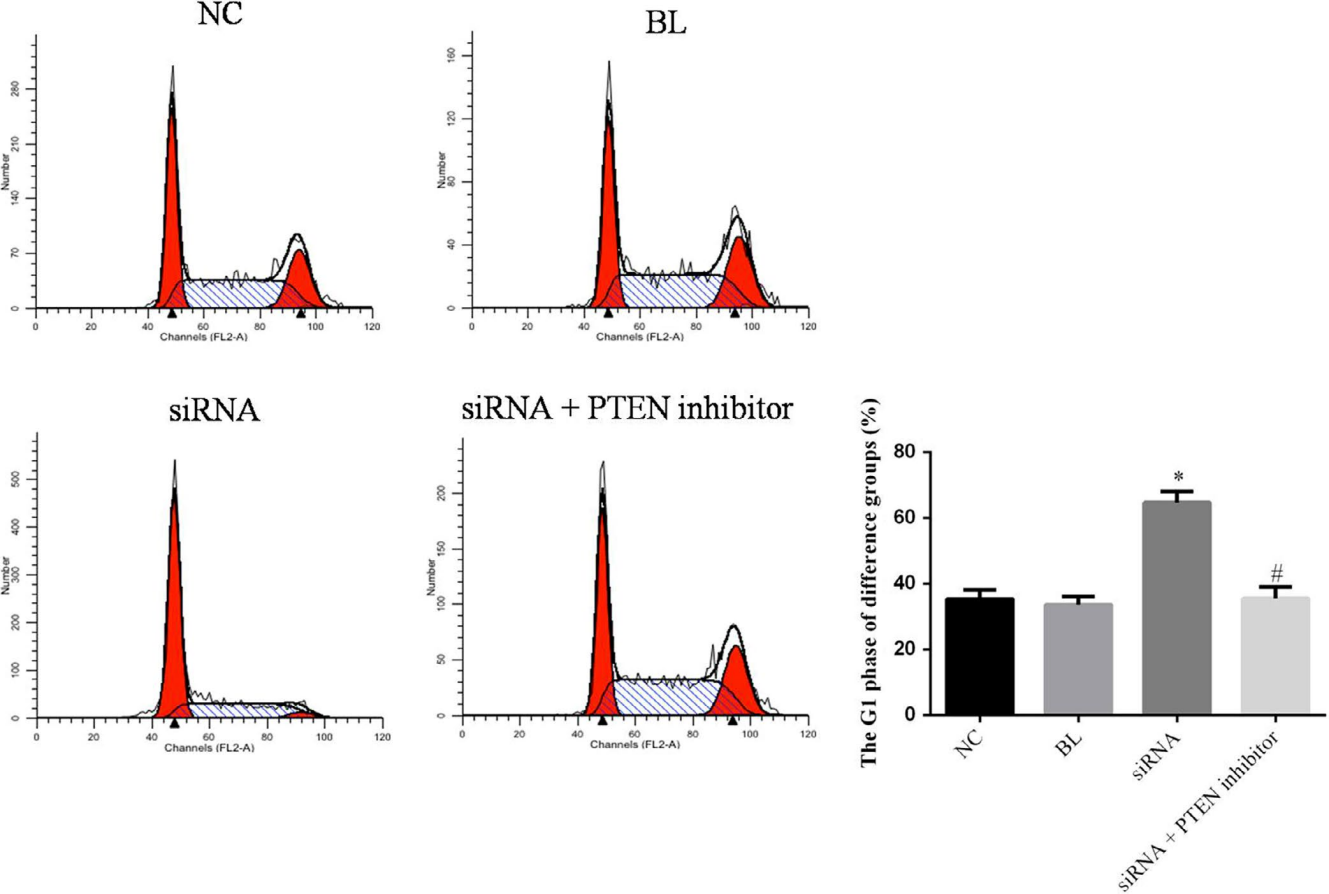
The cell cycle of difference group. *p < .05; compared with NC group. ^#^ *p* < .05; compared with simRNA group

Compared with NC group, the G1 phase rate of simRNA group was significantly enhanced (*p* < .05), and the simRNA+PTEN inhibitor group was no significantly differences (*p* > .05). The G1 phase rate of simRNA+PTEN inhibitor group was significantly suppressed compared with siRNA group (*p* < .05). The relative data are shown in Figure 4.

### 3.5 The simRNA has effects to cell invasion

**FIGURE 5:**
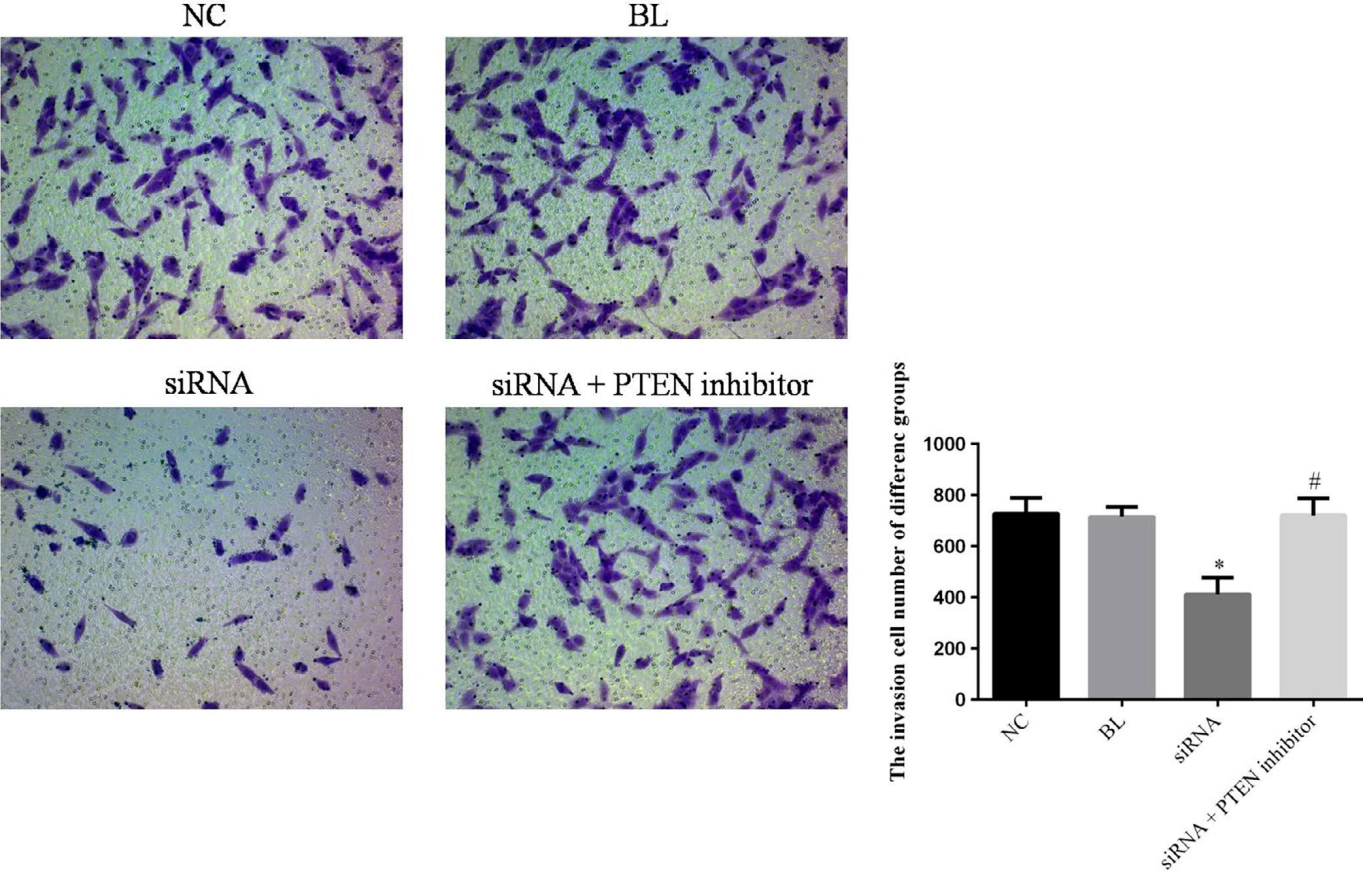
The cell invasion of difference groups by transwell. *p < .05; compared with NC group. ^#^ *p* < .05; compared with simRNA group

The invasion MG63 cell of simRNA group was significantly inhibited compared with NC groups with simRNA transfection (*p* < .05). The invasion MG63 cell number of simRNA+PTEN inhibitor group was significantly up‐regulated compared with simRNA group (*p* < .05) and was no significantly differences compared with NC group (*p* < .05). The relative data are shown in Figure 5.

### 3.6 The wound healing rate of difference groups

**FIGURE 6:**
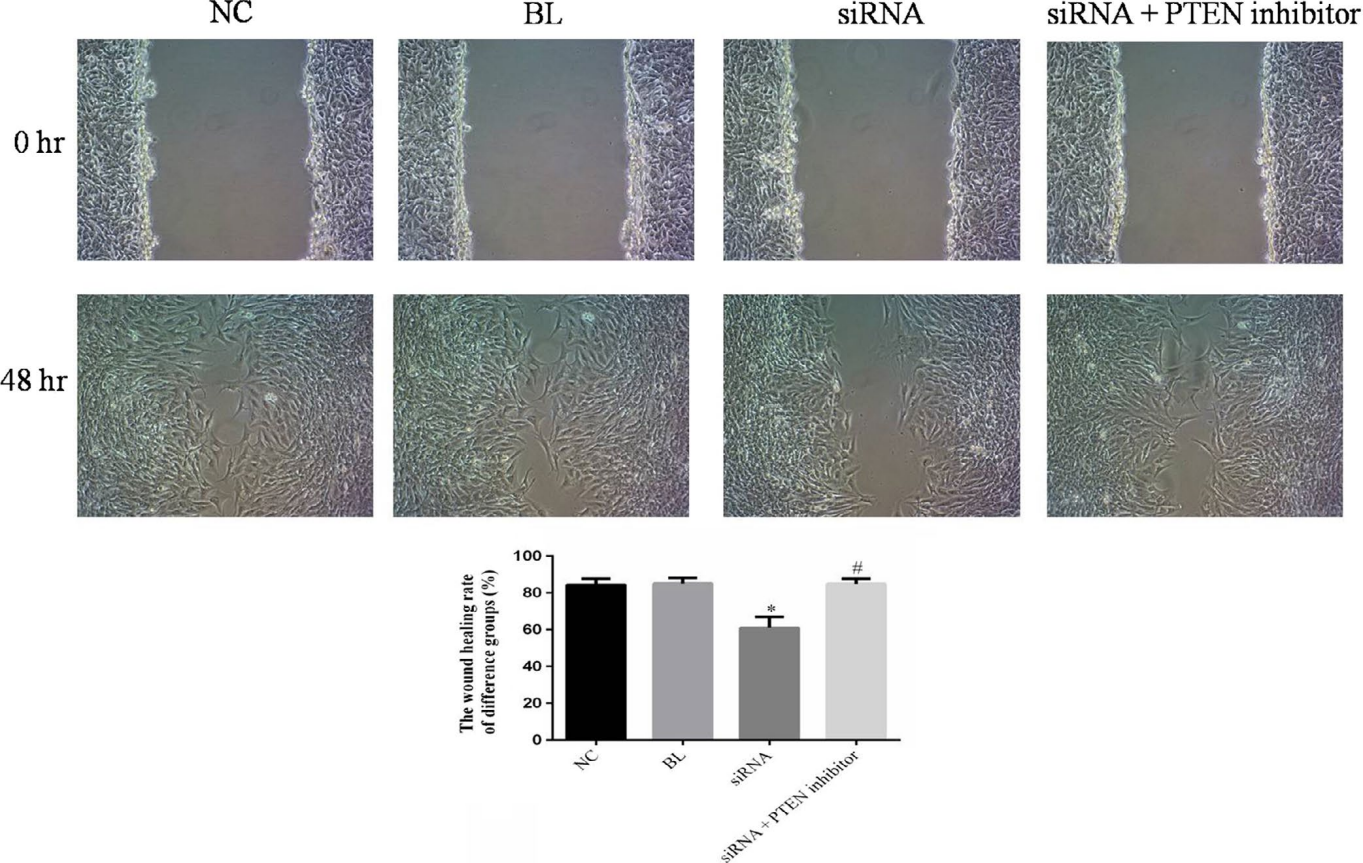
The wound healing rate of difference groups. *p < .05; compared with NC group. ^#^ *p* < .05; compared with simRNA group

The wound healing rate of simRNA group was significantly down‐regulated compared with NC group (*p* < .05). Meanwhile, the wound healing rate of simRAN+PTEN inhibitor group was significantly improved compared with simRNA group (*p* < .05) and no significantly differences compared with NC group (*p* > .05). The relative data are shown in Figure 6.

### 3.7 The simRNA has effects to relative proteins by WB assay

**FIGURE 7:**
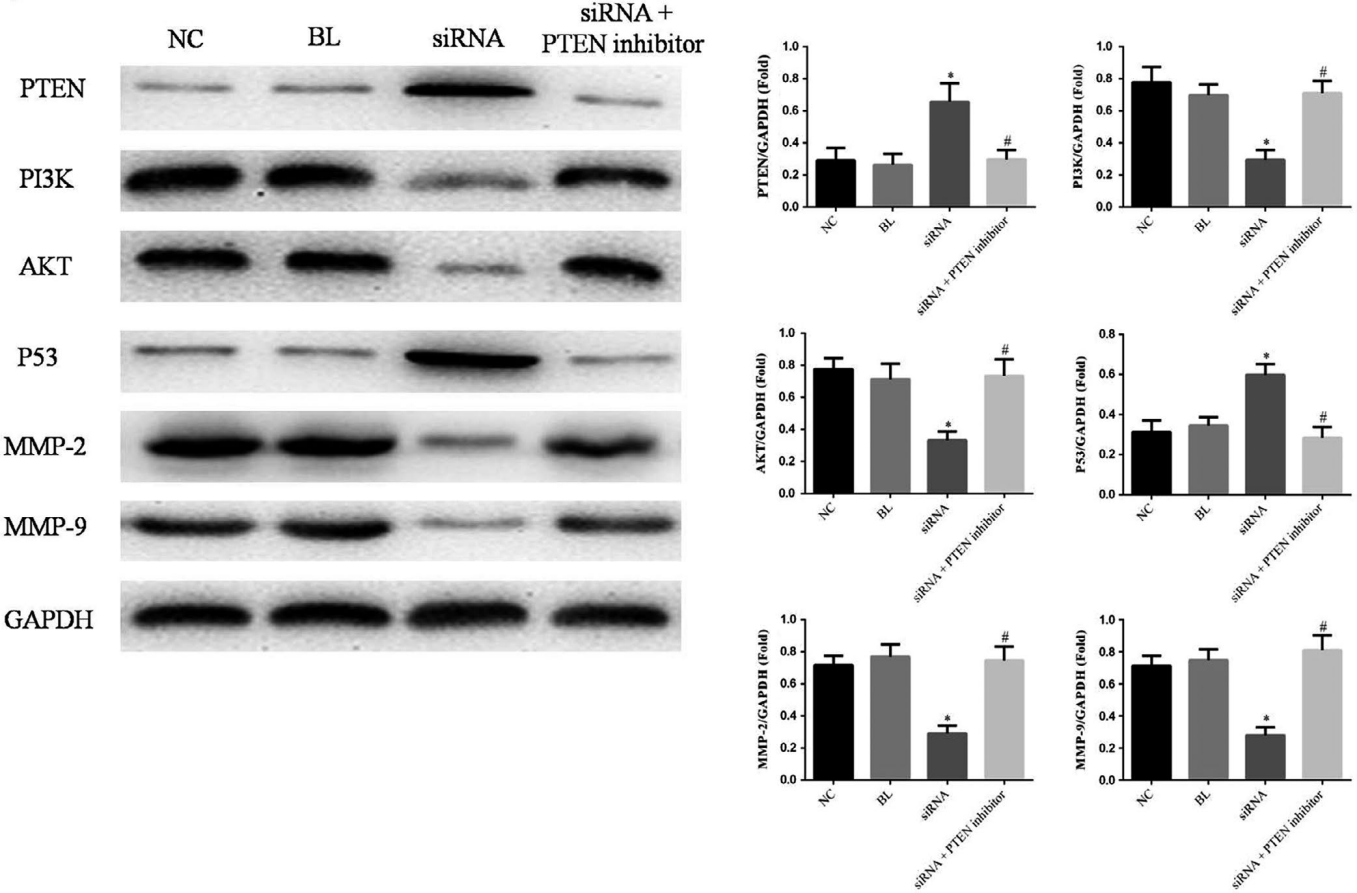
The relative proteins expressions by WB assay. *p < .05; compared with NC group. ^#^ *p* < .05; compared with simRNA group

Compared with NC group, the PTEN and P53 proteins expression of siRNA group were significantly enhanced and the PI3K, AKT, MMP‐2, and MMP‐9 protein expression were significantly suppressed (*p* < .05, respectively); However, the relative proteins expressions of simRNA+PTEN inhibitor group were no significantly differences. Compared with simRNA group, the relative protein expressions (PTEN, PI3K, AKT, P53, MMP‐2, and MMP‐9) were significantly differences (*p* < .05). The relative data are shown in Figure 7.

### 3.8 Correlation between miRNA‐216 and PTEN

**FIGURE 8:**
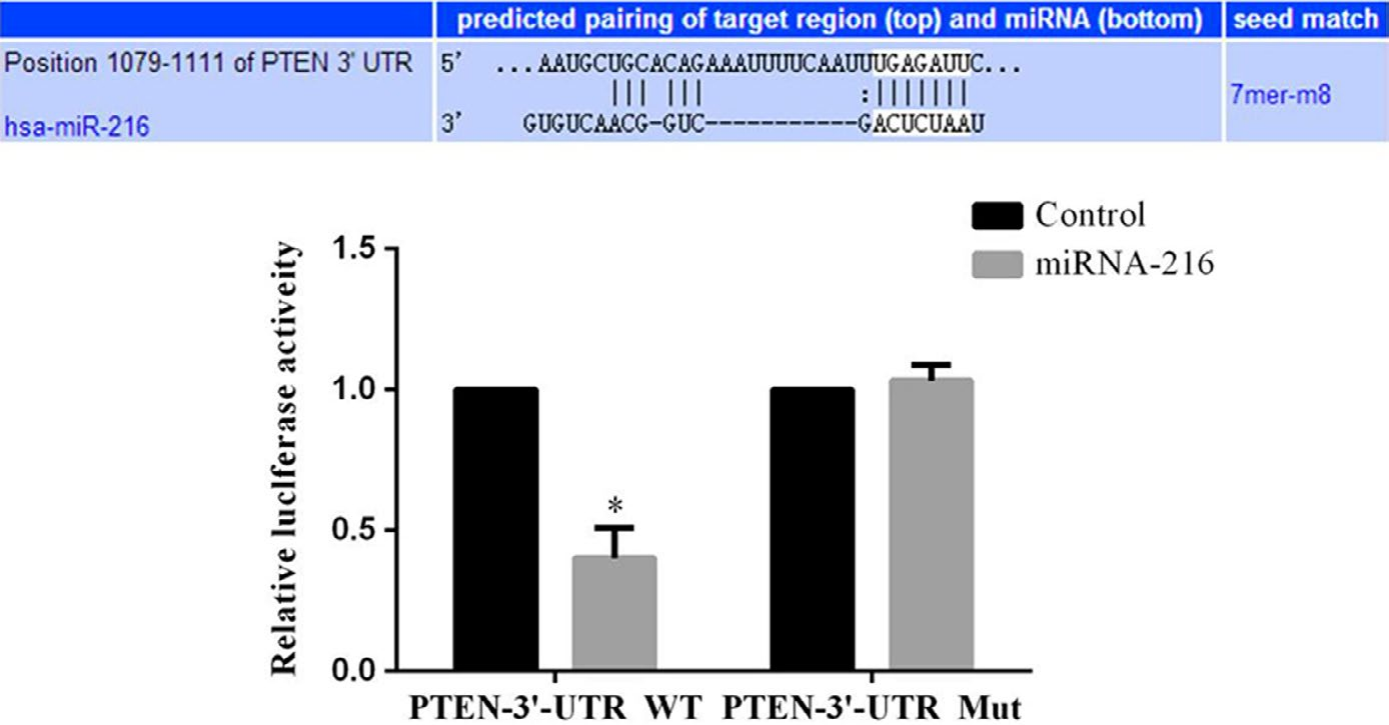
Dual luciferase assay. *p < .05; compared with control group

By dual luciferase assay, the results were shown that the miRNA‐216 overexpression had effects to suppress PTEN expression in PTEN‐3′‐UTR WT (*p* < .05). This result was confirmed that PTEN was the target gene of miRNA‐216. The relative data are shown in Figure 8.

## 4 DISCUSSION

PTEN was also reported in 1997 by three independent laboratories (Li & Sun, 1997; Li et al., 1997; Steck et al., 1997), in the following studies, the results were found that the change of PTEN activity is closely related to the occurrence of various types of tumors (Hsieh et al., 2000; Zhou et al., 2000). In our present clinical study, we found that PTEN protein expression was significantly reduced in the osteosarcoma tumor tissues. Depending on biological software analysis, we surmised that miRNA‐216 was targeted to the PTEN.

miRNAs are abnormal expression in a variety of cancers, so the research on the molecular mechanism of more and more attention, especially for the high incidence of juvenile osteosarcoma and poor prognosis of cancer, miRNA can provide clues for the study of disease occurrence and development mechanism and suggest new therapeutic targets. miRNA can promote cancer or tumor suppression in osteosarcoma, which is mainly determined by its downstream target genes (Varshney & Subramanian, 2015; Zhang et al., 2015). Like as, the up‐regulation of miR‐221 and miR‐199b‐5p in osteosarcoma is the role of promoting cancer, while miR‐503 and miR217 are down‐regulated in the tissue, mainly for tumor suppression (Chong et al., 2014; Wei, Deng, & Su, 2015; Zeng et al., 2016; Zhao et al., 2013). In our present study, we found that miRNA‐216 knockdown had effects to suppress MG63 cell proliferation, invasion, and migration and improve MG63 cell apoptosis by keeping in G1 phase.

In molecular biology level, we found that PI3K, AKT, P53, MMP‐2, and MMP‐9 proteins expressions were regulated with miRNA‐216 down‐regulation in our present study. PI3k is a cytosolic phosphatidylinositol kinase found by Sugimoto and Maeara and is associated with cancer products such as v‐src and v‐ras, which can specifically induce phosphorylation of the 3′ site on inositol rings. Akt is a serine/threonine protein kinase that is homologous with protein kinase C (73% homology) and protein kinase A (68% homology), also known as protein kinase B (PKB) (Xu et al., 2017). PI3k/Akt pathway plays a key role in cell homeostasis, neural development, metabolism and other processes. It regulates all aspects of cell development, such as apoptosis, cell cycle progression, and cell differentiation (Jin et al., 2017). The PI3k/Akt signaling pathway of growth factor receptor downstream substrate of PI3k and Akt anti‐apoptotic mechanism of scholar's ability to inhibit apoptosis, activation of Akt apoptosis by direct phosphorylation or indirectly change the encoding machine components of machine components apoptosis gene expression level, to control the apoptosis process (Chang et al., 2013; Moon et al., 2013; Yaoi et al., 2017). PI3k/Akt pathway can regulate cell growth, promote cell cycle proliferation and participate in angiogenesis. It is the most important signal pathway to inhibit apoptosis (Pfeifer et al., 2013). P53 was an important tumor‐suppressor gene which was a downstream gene of PI3K/AKT to keep cell cycle in G1 phase (Qiu, Leibowitz, Zhang, & Yu, 2010). Matrix metalloproteinases (MMPs), especially MMP‐2 and MMP‐9, play important roles in the decomposition of extracellular matrix (ECM) (Chen et al., 2013; Folgueras, Pendás, Sánchez, & López‐Otín, 2004). MMP‐2/9 can decompose most of the substance of ECM, which is beneficial for tumor cells to infiltrate into surrounding normal tissues, and promote tumor proliferation and metastasis. It is an important protein in tumor invasive growth. MMP2/9 was also an important downstream gene of PTEN/PI3K/AKT pathway (Li et al., 2013; Zhu et al., 2017). By double luciferase assay, we confirmed that PTEN was the target gene of miRNA‐216.

In conclusion, depending on our present study, we inferred that miRNA‐216 knockdown had antitumor effects via PTEN/PI3K/AKT and relative downstream gene P53 and MMP‐2/9.

## CONFLICT OF INTEREST

None.

## ETHICAL APPROVAL AND CONSENT TO PARTICIPATE

This study is approved by relevant Ethics Committee. This study is also obtained the signed informed consent from all participants/patient.

## CONSENT FOR PUBLICATION

Written consent was obtained.

## Data Availability

The datasets used or analyzed during the current study are available from the corresponding author on reasonable request.
